# Demographic Profile and Work Stress of Nursing Professionals in Public Hospitals in Aracaju, Sergipe

**DOI:** 10.3390/healthcare13182347

**Published:** 2025-09-18

**Authors:** Tânia Pereira dos Santos, Jeane dos Santos Ferreira, Calliandra Maria de Souza Silva, Izabel Cristina Rodrigues da Silva, Rita de Cássia Coelho Almeida Akutsu

**Affiliations:** 1 Postgraduate Program in Nutrition Sciences (PPGCNUT), Federal University of Sergipe, São Cristóvão 49107-230, SE, Brazil; fxtania@yahoo.com.br (T.P.d.S.); rita.akutsu@gmail.com (R.d.C.C.A.A.); 2 Nutrition Department, Federal University of Sergipe, São Cristóvão 49107-230, SE, Brazil; jeane.nut@hotmail.com; 3 Postgraduate Program in Health Science and Technology, University of Brasília (UnB), Brasília 70910-900, DF, Brazil; cdssilva@gmail.com

**Keywords:** health profile, occupational stress, nursing, healthcare workforce, Perceived Stress Scale (PSS-10), Brazilian Unified Public Health System (SUS)

## Abstract

**Background:** Occupational or work-related stress remains a persistent challenge in nursing, often intensified by sociodemographic factors. In Brazil’s Northeast, particularly in Aracaju, Sergipe, public hospital nurses face unique stressors shaped by regional socioeconomic conditions. **Objective:** This cross-sectional exploratory study aimed to examine the relationship between perceived work-related stress and the sociodemographic profiles of nursing professionals in three public hospitals in Aracaju. **Methods:** Data were collected via an online questionnaire incorporating the Perceived Stress Scale (PSS-10) and sociodemographic items. **Results:** Among 440 participants—comprising nurses (42%), nursing technicians (38.2%), nursing assistants (8.9%), and specialists (10.9%)—moderate to high stress levels were most prevalent among nursing technicians and assistants. Elevated stress was notably associated with adult women working in high-complexity sectors (e.g., pediatrics, obstetrics, ICU), particularly those without partners, earning low incomes, and with over ten years of professional experience. **Conclusions**: The findings highlight a vulnerable subgroup within the nursing workforce and underscore the need for targeted interventions to mitigate occupational stress in public healthcare settings. This study contributes region-specific insights into the intersection of stress and sociodemographic factors, offering a foundation for future policy and support strategies.

## 1. Introduction

The contemporary work scenario, characterized by constant economic and social transformations at both global and national levels, has redefined the relationships among individuals, organizations, and nations [1]. In the context of growing urbanization, where population concentration in large centers increases social demands [2,3], work emerges as a central theme for understanding the dynamics of contemporary life [4].

Historically, the concept of work has evolved from a connotation of suffering and punishment [3,5] to a fundamental cultural, social, psychological, and political value in post-industrial revolution society [6]. Work, alongside family, constitutes one of the most demanding dimensions of life for individuals, serving as both a source of production and meaning [7] and a potential source of discomfort [3]. Crucially, beyond its symbolic and existential dimensions, work remains a primary means of securing income, an essential factor for survival, autonomy, and social inclusion. The economic imperative to earn a living often shapes individuals’ choices and experiences in the workplace, especially in contexts marked by inequality and limited opportunities.

Nursing practice, while universal in its commitment to humanized care, manifests through distinct structural models across nations. In Brazil, the nursing workforce is organized into three regulated categories: nursing assistants, nursing technicians, and registered nurses [8,9,10,11]. These differences extend beyond nomenclature to encompass variations in training, scope of practice, and professional autonomy (Table 1). Despite the academic rigor required for Brazilian nurses, their professional autonomy is often constrained by a physician-centered model of care [8,9,10,11]. Understanding these distinctions is crucial for accurately interpreting the roles and responsibilities of Brazilian nursing professionals within the context of this study.

The World Health Organization (WHO) recognizes occupational stress as a growing global concern [12], defining it as the body’s response to external demands that require physical, mental, or emotional adjustments [13,14]. In the workplace, stress can be triggered by real or anticipated events, resulting in complex physiological reactions [12,15]. Prolonged exposure to chronic stress can have deleterious effects on the physical and mental health [16] of professionals, including health professionals.

Building on the WHO’s recognition of occupational stress, it is essential to contextualize the Brazilian experience within a broader global framework. This issue extends beyond nursing, affecting the entire healthcare sector. Doctors, physical therapists, technicians, and other professionals in the field also face a high risk of occupational stress. Its severity and impact are recognized worldwide and manifest in various forms, including professional absence and compromised quality of care. Although the data cited by Fiocruz (2021), which indicates a 47% rate of absences due to severe stress among Brazilian professionals in various healthcare fields, is relevant and alarming, it reflects a trend observed in other countries [17]. For example, studies conducted in nations such as Spain, the United Kingdom, and the United States also document high rates of stress among recent nursing graduates [18,19], and burnout, mental health problems and gastrointestinal disorders among nurses [20]. This global evidence supports the notion that stress is an inherent concern of the profession [21], driven by prolonged work hours, patient overload, and exposure to emotionally charged situations [20].

While occupational stress affects all healthcare professionals, nurses are particularly vulnerable due to the emotionally intense and physically demanding nature of their work. Recent studies confirm that burnout among nurses is not only widespread but escalating [22,23,24,25,26]. A 2025 umbrella review found that emotional exhaustion affects over 33% of nurses globally, with rates rising to 39% during the COVID-19 pandemic [24]. Sullivan et al. (2022) emphasize that burnout is a chronic stressor with profound consequences for mental and physical health, including increased risk of cardiovascular disease, sleep disturbances, and immune dysfunction [23]. These stressors—ranging from role conflict and moral distress to workplace violence and lack of team support—impact not only the well-being of professionals but also patient safety, job satisfaction, and retention. Addressing occupational stress in nursing is, therefore, a global imperative that requires systemic interventions, organizational accountability, and resilience-building strategies.

In Brazil, this issue is particularly pronounced within the Brazilian Unified Public Health System (Sistema Único de Saúde—SUS), one of the largest public health systems in the world [3,15,27]. Established by the 1988 Federal Constitution and guided by principles of universality, equity, and integrality, SUS provides free healthcare to over 75% of the Brazilian population, performing more than 11 million procedures daily [3]. It spans all 27 states and 5570 municipalities, encompassing primary care units, emergency services, hospitals, and specialized programs [3]. Nurses form the backbone of this system, representing over half of the health workforce and operating in every corner of the country—from urban centers to remote rural areas.

Despite its scale and achievements, SUS faces chronic underfunding, infrastructure gaps, and workforce shortages [3,15,27,28]. Occupational stress among Brazilian healthcare workers ranges from 27% to 87.4%, driven by factors such as work overload, limited resources, and poor management [28], with 65.9% of nursing professionals reporting occupational exhaustion [3], and over 56% required medical attention in the past year due to work-related health issues [3]. These stressors are compounded by low wages, precarious employment conditions, and exposure to workplace violence, which exacerbate stress levels [3,15]. While these challenges are present nationwide, regional disparities make certain areas particularly vulnerable to them. In Aracaju, Sergipe (in the Brazilian Northeast Region), these challenges are intensified by regional disparities in resource allocation and professional distribution, making it a critical site for examining the intersection of stress, socioeconomic factors, and professional well-being.

Given this backdrop, both globally and within the Brazilian SUS, the present study analyzes the relationships between socioeconomic and demographic characteristics and the perception of occupational stress among nursing professionals in public hospitals in Aracaju, Sergipe. By identifying the social and economic factors that exacerbate psychological stress, the study supports the development of targeted interventions and health policies that promote nurses’ physical and mental well-being, contributing to the valorization of the profession and the improvement of working conditions within the universal healthcare system.

## 2. Materials and Methods

### 2.1. Study Design and Setting

This cross-sectional, exploratory study was conducted with nursing professionals working in three public hospitals located in Aracaju, Sergipe, Brazil. These hospitals are administered by Sergipe’s State Health Department and are referred to as Hospital 1, Hospital 2, and Hospital 3 to maintain confidentiality for both the institutions and the participants. Data collection occurred between September and December 2024 and was carried out in sequential stages.

### 2.2. Ethical Considerations

The study was approved by the Research Ethics Committee (CEP) of the Federal University of Sergipe (approval no. 80679524.0.0000.5546), in accordance with ethical and legal standards. All participants provided informed consent by signing a printed Free and Informed Consent Form (FICF), as required by the ethics committee. Participants were informed that they could withdraw their authorization for data use at any time, and this information was reiterated during recruitment.

### 2.3. Participants and Recruitment

Participants were selected through convenience sampling. Eligible individuals included nursing professionals actively working in one of the three hospitals who voluntarily agreed to participate and signed the FICF. Professionals not engaged in direct patient care and those with cognitive impairments that could hinder comprehension of the instruments were excluded. The hospitals’ Teaching and Research Centers (NEP—Núcleos de Ensino e Pesquisa) were contacted to authorize and publicize the study. After approval, researchers visited the hospitals and recruited participants in person by approaching them in their work areas—such as nursing stations, cafeterias, reception areas, and nursing rooms. Only professionals available at the time and directly involved in patient care were invited to participate. According to NEP records, individuals with mental health diagnoses were removed from patient-facing roles and thus were not accessible to the researchers.

### 2.4. Data Collection Procedure

Data collection was conducted in two stages. First, researchers engaged nursing professionals in collective or individual conversations to present the study objectives and methodology. In the second stage, participants who agreed to participate signed the printed FICF. After consent was obtained, researchers provided a QR code that linked to a Google Forms questionnaire. The form was completed digitally and included mandatory questions covering personal and social data, the Perceived Stress Scale (PSS-10), and the Body Image Perception Scale (Brazilian Silhouette Scale).

The study strictly followed approved ethical protocols. In keeping with the cross-sectional methodology employed, stress levels were identified at a single point in time, without longitudinal monitoring or intervention. Although the study did not aim for direct intervention, the ICF informed participants that if they felt uncomfortable with the topic or the results, they could seek psychological support through the hospital’s mental health network or other available services. This approach ensured that participants were aware of their rights and the resources available to them.

Additionally, the data provided by the human resources departments of each institution included not only the total number of nursing professionals but also those who were on leave during the data collection period. This detail was important for understanding workforce dynamics and contextualizing participation rates.

### 2.5. Survey Instruments

The study used two instruments for data collection. The first was a Sociodemographic Questionnaire developed by the authors, which gathered information on self-declared gender, age, professional role, educational level, family income, religion, sector of activity (specialty), and job tenure. These variables were defined according to the standards of the Brazilian Institute of Statistics [2] and the Federal Council of Nursing [29]. The second instrument was the Perceived Stress Scale (PSS-10), a validated Brazilian version translated and adapted by Luft et al. [30] from the original scale by Cohen et al. [31]. The PSS-10 consists of 10 items that assess the frequency of stress-related feelings and thoughts over the past month, using a five-point Likert scale ranging from 0 (never) to 4 (very often). Total scores range from 0 to 100 and are categorized as follows: 0–25 (low or mild stress), 26–50 (moderate stress), 51–75 (high stress), and 76–100 (very high stress). Scores equal to or greater than 40 are considered indicative of chronic stress and elevated health risk [30,31].

The Sociodemographic Questionnaire, developed by the authors, collected data on self-declared gender, age, educational level, family income, religion, sector of activity (specialty), job tenure, and professional function. In this study, “function” refers to the professional practice and duties assigned according to hierarchical position (Table 1).

### 2.6. Statistical Analysis

Data analysis was performed using SPSS^®^ version 29.0. Descriptive statistics were used to characterize the sample, with categorical variables presented as absolute and relative frequencies, and continuous variables summarized using means and standard deviations or medians and percentiles when data were not normally distributed. Associations between stress levels and sociodemographic or functional variables were analyzed using the ANOVA with Tukey’s post hoc test (parametric test) or Mann–Whitney U test, Spearman’s correlation, and the chi-square test (for data were not normally distributed). All statistical tests adopted a significance level of α = 0.05 (two-tailed) and a 95% confidence interval [32].

## 3. Results

### 3.1. Sociodemographic and Economic Characteristics

Table 2 presents the sociodemographic and professional characteristics of the 440 nursing professionals who participated in the study. Participants were recruited from three public hospitals in Aracaju and included both nurses and nursing technicians or assistants. The table summarizes key variables such as gender, age, race, marital status, religion, income, professional training, and years of experience.

### 3.2. Perceived Work-Related Stress

#### 3.2.1. Self-Declared Gender and Stress Levels

Among women, 81.66% (n = 334) reported moderate stress, 9.29% (n = 38) mild stress, and 9.05% (n = 37) high stress. Among men, 61.29% (n = 19) reported moderate stress, 35.48% (n = 11) mild stress, and 3.23% (n = 1) high stress. The median stress score for females was 20.00 (P25 = 17.00; P75 = 23.00), and for males, 18.00 (P25 = 12.00; P75 = 22.00). Although no inferential tests were applied, the difference in medians suggests a potential trend toward higher stress among women. A chi-square test of independence was conducted to examine the association between sex and stress level categories (mild, moderate, and high). The results revealed a statistically significant association, χ^2^ (2, N = 440) = 20.37, *p* < 0.001, indicating that the distribution of stress levels differed between women and men. Specifically, women reported a higher proportion of moderate and high stress compared to men, who more frequently reported mild stress. The effect size, measured by Cramér’s V (0.215), suggested a small-to-moderate strength of association.

#### 3.2.2. Age and Stress Levels

Among the adults evaluated, 77.5% (n = 341) reported moderate levels of stress, 10.7% (n = 47) reported mild stress, and 8.6% (n = 38) reported high stress. In contrast, within the subgroup of older adults (aged over 60 years), 2.7% (n = 12) reported moderate stress, 0.45% (n = 2) reported mild stress, and no cases of high stress were observed. These findings indicate that moderate stress is prevalent across different age groups, whereas high stress appears to be less frequent among older professionals. Otherwise, the relationship between age (considered in single-year intervals) and categorical stress levels (mild, moderate, high) was examined through a contingency table analysis. The Pearson chi-square test was applied to determine the presence of an association between these variables. The test returned a chi-square statistic of χ^2^ = 59.332 with 58 degrees of freedom and a corresponding significance value of *p* = 0.427, indicating the absence of a statistically significant association.

On the other hand, to evaluate the relationship between age and mean stress level, Spearman’s rank-order correlation was employed (the analysis yielded a correlation coefficient of Spearman = −0.033, with a two-tailed significance level of = 0.862). This result indicates no statistically significant correlation between age and mean stress level, suggesting that variations in age do not have a substantial impact on the perceived level of stress among participants.

#### 3.2.3. Self-Declared Race and Stress Levels

Although differences in average stress levels were observed among racial groups, these did not reach statistical significance (ANOVA: F (3, 436) = 2.194, *p* = 0.088). Thus, self-declared race was not a significant predictor of perceived work-related stress.

#### 3.2.4. Marital Status and Stress Levels

Regarding marital status, 67.0% (n = 295) reported not having a partner, 32.5% (n = 143) had a partner, and 0.7% (n = 2) did not report. Statistical analysis using the chi-squared test revealed that *p* < 0.0001, so the distribution of marital status percentages in the different stress levels was statistically significant.

#### 3.2.5. Religion and Stress Levels

The overall mean stress score was 19.92 (SD = 5.23; scores ranging from 4 to 38), with subgroup means ranging from 18.84 (for those who did not report a religion) to 20.07 (for evangelicals). Tukey’s post hoc test revealed no statistically significant differences between groups. ANOVA results for religious affiliation also indicated no significant associations with perceived stress (total stress: F (3, 436) = 0.703, *p* = 0.551; stress assessment: F (3, 436) = 1.147, *p* = 0.330).

#### 3.2.6. Professional Role and Stress Levels

Using the Chi-square, no significant differences were found in stress levels among nurses, nursing technicians, and assistants (*p* = 0.884). This non-significance suggests a homogenous perception of stress across different roles or functions. Table 3 summarizes stress levels by professional role.

#### 3.2.7. Work Sector and Stress Levels

A statistically significant difference was found in perceived stress across hospital sectors (Chi-square test, *p* = 0.004). Higher stress levels were observed in professionals working in the Pediatric ICU, obstetric emergency, and obstetric hospitalization sectors. Table 4 presents the stress levels by sector.

#### 3.2.8. Experience, Salary, and Stress Levels

No statistically significant associations were found between stress and time in the role/job tenure (*p* = 0.425) or salary level (*p* = 0.648), indicating that neither variable predicted stress in the studied sample.

### 3.3. Summary of Perceived Work-Related Stress Levels

The average perceived stress score was 19.92 (SD = 5.23), with a median of 20.00. The interquartile range (P25 = 17.00; P75 = 23.00) indicates that half of the sample scored within this range. Scores varied from a minimum of 4.00 to a maximum of 40.00.

## 4. Discussion

The study examined the relationship between the sociodemographic and economic characteristics of nursing professionals and their levels of perceived workplace stress. The findings reaffirm that stress in nursing is a multifactorial phenomenon, shaped not only by financial conditions but also by gender, race, professional role, and work environment.

A higher incidence of perceived stress was observed among women, corroborating findings from other studies [33]. This gender vulnerability is compounded by societal expectations, such as aesthetic standards, double or triple work shifts, and the burden of unpaid care work (caring for others and fulfilling domestic responsibilities), and institutional devaluation contributes to demotivation and stress [33,34]. These findings are consistent with international literature, which shows that female healthcare workers report significantly higher levels of emotional exhaustion and anxiety than their male counterparts due to gendered labor divisions and systemic undervaluation of care work [35,36].

In contrast, male professionals presented substantially lower levels of stress [2,37], reinforcing the gender gap in emotional burden within nursing. Regarding age, young adults were more susceptible to moderate stress and mental overload, while older professionals demonstrated greater resilience. However, prolonged exposure to stressors may lead to chronic stress, a pattern observed in international studies on ICU nurses, where burnout prevalence ranges from 25% to 45% [38,39,40].

The predominance of professionals who self-identified as mixed race reflects the demographic composition of Sergipe [41,42]. Although the study did not identify race as a predictor of stress, the choice of some professionals not to declare their color may reflect structural racism, indicating insecurity in work environments perceived as hostile or of racial ambiguity that masks inequalities. This aligns with national studies that highlight racial ambiguity and institutional silence around race as mechanisms that mask inequalities in Brazilian healthcare settings [11]. Internationally, Tembo et al. (2025) [43] and Hamed et al. (2022) [44] show that racialized nurses often experience marginalization, invisibility, and hyper-surveillance in clinical environments, contributing to stress and professional dissatisfaction.

The presence of a partner was shown to be a protective factor against perceived stress, highlighting the importance of social support in managing work demands. Professionals with a partner may benefit from emotional and practical support, while those without such support may face a greater overload of responsibilities. This is supported by the COVID-19 HEalth CaRe WOrkErs Study (HEROES), which found that healthcare workers with stronger social networks reported lower psychological distress during the COVID-19 pandemic, which found that healthcare workers with stronger social networks reported lower psychological distress during the COVID-19 pandemic [45].

Religiosity was not a major predictor of stress in this study. However, certain practices and belief systems may provide coping mechanisms, such as managing and resilience, suggesting that the way faith is experienced may act as a protective factor. Sullivan et al. (2022) found that spiritual well-being reduced emotional exhaustion and depersonalization among nurses, reinforcing the potential role of spirituality in stress mitigation [22,23].

The study confirmed that moderate stress is a prolonged state of alert with potential adverse effects, and its perception is subjective, varying among professionals [46,47]. The analysis of the function and job tenure did not identify a statistical difference in the perception of stress, suggesting that stress in nursing is a transversal experience related to multiple areas beyond direct care.

The nursing professional’s area of activity (specialty) was shown to be the primary determinant of the perception of work-related stress. Environments such as intensive care units (ICUs) and obstetrics and pediatrics sectors presented the highest levels of stress, attributed to the high complexity, severity of cases and intense emotional demand. These findings are consistent with international evidence that ICU and pediatric nurses face elevated moral distress, emotional trauma, and burnout [48,49]. In contrast, sectors such as pediatric emergency and central sterile material (CME) presented lower averages. These findings underscore the importance of stress management and mitigation strategies tailored to the unique characteristics of each environment.

The hospital environment can reproduce broader social dynamics. The need to constantly prove oneself, the feeling of being underestimated, or barriers to career advancement can be sources of stress that are not explicitly measured by average scores but contribute to psychological exhaustion, as evidenced by Silva and Bendassoli [50]. These dynamics are echoed in global studies that highlight how institutional cultures and leadership structures can perpetuate stress through lack of recognition and limited career mobility [35].

The consequences of stress go beyond the psychological aspect, manifesting themselves in both a somatic and systemic way, contributing to cardiovascular diseases, gastrointestinal disorders, fatigue and weakening of the immune system [51]. In addition to the physical impact, stress affects mental health, increasing the risk of anxiety, depression and irritability, which compromises professional performance and quality of care and contributes to absenteeism and presenteeism (low work efficiency) [51]. Sullivan et al. (2022) emphasize that burnout disrupts immune and neuroendocrine function, increasing vulnerability to illness and emotional instability [22,23].

Interestingly, income was unassociated with perceived stress in this study. This lack of association suggests that the main stressors in nursing are intrinsic to the work, such as the intensity of the workday, working conditions, emotional pressure, and relationships with staff and patients, outweighing the direct impact of the financial factor. For these professionals, salary satisfaction, although important, does not automatically translate into less work stress. Similar findings were reported in the Frontiers in Global Women’s Health review, which identified systemic stress triggers beyond salary, including lack of recognition and high emotional labor [35].

## 5. Study Limitations and Contextual Contributions

The methodology adopted for this study proved adequate for data collection; however, some contextual and relational limitations emerged and deserve consideration. The main one lies in the resistance of some professionals to participate, which, although it may have generated selection bias, constituted a significant contribution to the research. This resistance manifested itself not only due to time constraints, but also through explicit objections from participants.

The dialogue established with the professionals, which clarified the purpose of the research and the dynamics of accessing the form, revealed a deep sense of distrust. Some verbally expressed the belief that such research did not translate into tangible improvements for the nursing team, but rather solely benefited the administrative agenda. Additionally, the fear that exposing issues related to institutional stress could result in a negative perception of their professional image within the institution demonstrates their vulnerability and fear of retaliation.

Thus, the appropriate methodology allowed the context itself and the dialogue established with participants to become valuable sources of information. The refusal to fill out online forms and the comments expressed about distrust of research are, in themselves, qualitative data that reveal an immeasurable level of stress and a complex reality that cannot be captured at that time solely through questionnaires. These factors highlight the importance of an ethical and careful approach to research in healthcare settings.

Another important limitation is that the research was conducted in only three public hospitals in Aracaju. The lack of private institutions or hospitals in other locations in the country limits the ability to generalize the findings to all nursing professionals in Brazil. Therefore, the results obtained are specific to the context analyzed and may not reflect the reality of other work environments with different management profiles, workloads, and resources.

## 6. Conclusions

This study identified key sociodemographic and economic factors—such as professional role, income level, marital status, and years of experience—that significantly influence psychological work-related stress among nursing professionals in public hospitals in Aracaju, Sergipe. While variables like race and religious affiliation showed limited predictive value, the presence of a partner and working in less complex sectors appeared to mitigate stress levels.

The findings confirm that nursing technicians and assistants, particularly adult single women with low income and long job tenure in high-complexity units, represent a vulnerable group. These insights directly support the study’s objective by providing evidence for targeted interventions and informing health policies aimed at promoting mental well-being and improving working conditions.

To address these challenges, the Brazilian Unified Health System (SUS) needs to prioritize psychosocial support, ensure equitable access to mental health resources, and tailor stress prevention strategies to the diverse needs of nursing professionals. These measures will contribute to the valorization of the nursing workforce and enhance the sustainability of care within the universal healthcare system.

## Figures and Tables

**Table 1:** Structure of the Brazilian Nursing Workforce [8,9,10,11].

Category	Education Level	Scope of Practice	Regulatory Body
Nursing Assistant	Secondary-level vocational training	Provides basic patient care under supervision; assists with hygiene, feeding, mobility	Brazilian Federal Nursing Council (COFEN)
Nursing Technician	Post-secondary technical education	Performs more complex procedures; administers medications; supports clinical routines	COFEN and Regional Councils (CORENs)
Registered Nurse (RN)	University-level degree (Bachelor’s in Nursing)	Leads care planning; supervises assistants/technicians; engages in clinical decision-making	COFEN and CORENs

**Table 2:** Participants’ Sociodemographic and Professional Characteristics.

Variable	Category	N (Total = 440)	%
Hospital	Hospital #1	232	52.7%
	Hospital #2	44	10.0%
	Hospital #3	164	37.3%
Professional Role	Nurse	119	27.0%
	Technician/Assistant	321	73.0%
Professional Training	Nurse	185	42.0%
	Nursing Technician	168	38.2%
	Nursing Assistant	39	8.9%
	Specialization	48	10.9%
Gender	Female	409	93.0%
	Male/Other	31	7.0%
Age	Adults	426	96.8%
	Mean Age (SD)	—	42 (±8.2) years
Race	Mixed Race	191	43.4%
	Black	64	14.5%
	White	50	11.4%
	Not Reported	135	30.7%
Marital Status	Not in a Relationship	297	67.5%
	In a Relationship	143	32.5%
Religion	Catholic	258	58.6%
	Evangelical	106	24.1%
	Spiritualist	31	7.0%
	Not Specified	45	10.2%
Income	Up to 1 Minimum Wage ^$^	27	6.1%
	Up to 2 Minimum Wages	168	38.2%
	Up to 3 Minimum Wages	124	28.2%
	More than 3 Minimum Wages	62	14.1%
	Not Disclosed	59	13.4%
Experience	>10 Years	287	65.2%
	≤10 Years	153	34.8%

Source: Study data, 2024. Note: ^$^ Brazilian Minimum Wage is R$1518.00 (~USD$280).

**Table 3:** The relationship between function and work-related stress perceived by nursing professionals in public hospitals in Aracaju, Sergipe (2024).

Function	Mean Perceived Stress	Count (N)	Mild Stress % (n)	Moderate Stress % (n)	High Stress % (n)
Nursing Assistant	20.29	12.5% (55)	10.9% (6)	80% (44)	9.1% (5)
Nurse	20.16	27.0% (119)	7.6% (9)	84% (100)	8.4% (10)
Nursing Technician	19.73	60% (264)	12.9% (34)	78.4% (207)	8.7% (23)
Preferred not to inform	20.00	0.45 (2)	0.0%	100.0% (2)	0.0%

Source: Study data, 2024.

**Table 4:** Perceived work-related stress levels among nursing professionals in public hospitals in Aracaju, Sergipe, according to their activity sector.

Hospital Sector	Mean Perceived Stress	Count (N)	Mild Stress % (n)	Moderate Stress % (n)	High Stress % (n)
Adult hospitalization	2.01	156	12.2% (19)	80.8% (126)	7.1% (11)
Pediatric hospitalization	1.88	14	14.3% (2)	71.4% (10)	14.3% (2)
Obstetric hospitalization	2.12	18	0.0% (0)	88.9% (16)	11.1% (2)
Adult emergency	1.90	75	12.0% (9)	82.7% (62)	5.3% (4)
Pediatric emergency	1.72	11	18.2% (2)	81.8% (9)	0.0% (0)
Obstetric emergency	2.11	26	7.7% (2)	80.8% (21)	11.5% (3)
Surgical center	1.92	44	15.9% (7)	79.5% (35)	4.5% (2)
Administrative/Management	—	2	0.0% (0)	100.0% (2)	0.0% (0)
SMC— Sterilization Material Center	1.79	16	12.5% (2)	87.5% (14)	0.0% (0)
ICU—Adult	1.99	35	5.7% (2)	85.7% (30)	8.6% (3)
ICU—Pediatric	2.18	21	4.8% (1)	81.0% (17)	14.3% (3)
ICU—Neonatal	1.94	22	4.5% (1)	86.4% (19)	9.1% (3)

Source: Study data, 2024.

## Data Availability

Data is contained within the article.

## References

[1] Da Silva V.G.F., da Silva B.N., Pinto É.S.G., de Menezes R.M.P. (2021). The Nurse’s Work in the Context of COVID-19 Pandemic. Rev. Bras. Enferm..

[2] IBGE—Instituto Brasileiro de Geografia e Estatística Censo Brasileiro de 2022: Brasil, 2023. https://www.gov.br/secom/pt-br/assuntos/noticias/2023/06/censo-2022-indica-que-o-brasil-totaliza-203-milhoes-de-habitantes.

[3] Da Silva M.C.N., Machado M.H. (2020). Sistema de Saúde e Trabalho: Desafios Para a Enfermagem No Brasil. Cien. Saude Colet.

[4] Borges L.d.O. (2010). A Psicologia Do Trabalho e Das Organizações No Brasil Floresce?. Estud. Psicol..

[5] Albornoz S. (2017). O Que é Trabalho Primeiros Passos.

[6] Bendassolli P.F. (2017). Work and Culture: Approaching Cultural and Work Psychology. Cult. Psychol..

[7] Bendassolli P.F., Soboll L.A.P. (2011). Clínicas Do Trabalho: Filiações, Premissas e Desafios. Cad. Psicol. Soc. Trab..

[8] Machado M.H., Filho W.A., Lacerda W.F.d., Oliveira E., Lemos W., Wermelinger M., Vieira M., Santos M.R.d., de Souza Junior P.B., Justino E. (2016). Características Gerais Da Enfermagem: O Perfil Sociodemográfico. Enferm. Em Foco.

[9] Flinkman M., Isopahkala-Bouret U., Salanterä S. (2013). Young Registered Nurses’ Intention to Leave the Profession and Professional Turnover in Early Career: A Qualitative Case Study. ISRN Nurs..

[10] Barbosa L.R., Pereira L.L. (2015). Nursing Education in a Curriculum Oriented of Competence: A Systematic Review. Creat. Educ..

[11] Cesário J.M.d.S., Flauzino V.H.d.P., Hernandes L.d.O., Gomes D.M., Vitorino P.G.d.S. (2021). Prática Clínica Do Enfermeiro: Diferenças Entre Brasil E Estados Unidos Da América. Rev. Científica Multidiscip. Núcleo Conhecimento.

[12] Houtman I., Jettinghoff K., Cedillo L. (2007). Raising Awareness of Stress at Work in Developing Countries: A Modern Hazard in a Traditional Working Environment: Advice to Employers and Worker Representatives. WHO Libr. Cat. Publ. Data Prot. Work. Health Ser..

[13] Fink G. (2016). Stress, Definitions, Mechanisms, and Effects Outlined. Stress: Concepts, Cognition, Emotion, and Behavior.

[14] Eduardo L.d.S., Gurgel P.C., Da Silva B.N., Do Nascimento P.F.D., Lameira A.P.d.N., Flavio F.F. (2020). Análise Do Estresse Ocupacional Em Técnicos de Enfermagem: Correlação Entre Medidas Psicológica e Fisiológica. Enferm. Bras..

[15] Pires B.S.M., de Oliveira L.Z.F., Siqueira C.L., Feldman L.B., Oliveira R.A., Gasparino R.C. (2018). Nurse Work Environment: Comparison between Private and Public Hospitals. Einstein.

[16] Kumar S., Jain A.K. (2012). Essence and Consequences of Stress in the Workplace. J. Organ. Hum. Behav..

[17] Fundação Oswaldo Cruz (2022). Ministério da Saúde Anuário Estatístico de Saúde Do Trabalhador Fiocruz 2021.

[18] Merino-Godoy M.-Á., Aceijas Z.M., Martín M.C., Gago-Valiente F.-J., Abengozar A.V., Padilla J.M.P., da Costa E.T. (2024). Navigating Perceived Stress: Experiences of Nursing Students Completing Internships during the COVID-19 Pandemic in Spain. J. Clin. Med..

[19] Meneguin S., Pollo C.F., Segalla A.V.Z., Generoso F.J.F., de Leo A., de Oliveira C. (2024). Stress and Occupational Coping among Brazilian Nurses in Critical Care Units during the COVID-19 Pandemic. Healthcare.

[20] Roman P., Perez-Cayuela I., Gil-Hernández E., Rodriguez-Arrastia M., Aparicio-Mota A., Ropero-Padilla C., Rueda-Ruzafa L. (2023). Influence of Shift Work on The Health of Nursing Professionals. J. Pers. Med..

[21] Schneider-Matyka D., Świątoniowska-Lonc N., Polański J., Szkup M., Grochans E., Jankowska-Polańska B. (2023). Assessment of The Effect of Stress, Sociodemographic Variables and Work-Related Factors on Rationing of Nursing Care. Int. J. Environ. Res. Public Health.

[22] Sullivan V., Hughes V., Wilson D.R. (2022). Nursing Burnout and Its Impact on Health. Nurs. Clin. N. Am..

[23] Sullivan D., White K.M., Frazer C. (2022). Factors Associated with Burnout in the United States Versus International Nurses. Nurs. Clin. N. Am..

[24] Getie A., Ayenew T., Amlak B.T., Gedfew M., Edmealem A., Kebede W.M. (2025). Global Prevalence and Contributing Factors of Nurse Burnout: An Umbrella Review of Systematic Review and Meta-Analysis. BMC Nurs..

[25] Jun J., Ojemeni M.M., Kalamani R., Tong J., Crecelius M.L. (2021). Relationship between Nurse Burnout, Patient and Organizational Outcomes: Systematic Review. Int. J. Nurs. Stud..

[26] Okuhara M., Sato K., Kodama Y. (2021). The Nurses’ Occupational Stress Components and Outcomes, Findings from an Integrative Review. Nurs. Open..

[27] Patrício D.F., Barbosa S.d.C., da Silva R.P., da Silva R.F. (2022). Dimensões de Burnout Como Preditoras Da Tensão Emocional e Depressão Em Profissionais de Enfermagem Em Um Contexto Hospitalar. Cad. Saúde Coletiva.

[28] Lorenz V.R., Sabino M.O., Corrêa Filho H.R. (2018). Professional Exhaustion, Quality and Intentions among Family Health Nurses. Rev. Bras. Enferm..

[29] COFEN—Conselho Federal de Enfermagem Quantitativo de Profissionais Por Regional 2025. https://descentralizacao.cofen.gov.br/sistema_SC/grid_resumo_quantitativo_profissional_externo/.

[30] Luft C.D.B., Sanches S.d.O., Mazo G.Z., Andrade A. (2007). Versão Brasileira Da Escala de Estresse Percebido: Tradução e Validação Para Idosos. Rev. Saude Publica.

[31] Cohen S., Kamarck T., Mermelstein R. (1983). A Global Measure of Perceived Stress. J. Health Soc. Behav..

[32] Mishra P., Singh U., Pandey C.M., Mishra P., Pandey G. (2019). Application of Student’s t-Test, Analysis of Variance, and Covariance. Ann. Card. Anaesth..

[33] Rogers C.B., Webb J.B., Jafari N. (2018). A Systematic Review of the Roles of Body Image Flexibility as Correlate, Moderator, Mediator, and in Intervention Science (2011–2018). Body Image.

[34] COFEN—Conselho Federal de Enfermagem RESOLUÇÃO COFEN No 564/2017—Código de Ética Dos Profissionais de Enfermagem. https://www.cofen.gov.br/resolucao-cofen-no-5642017/.

[35] Sriharan A., Ratnapalan S., Tricco A.C., Lupea D., Ayala A.P., Pang H., Lee D.D. (2020). Occupational Stress, Burnout, and Depression in Women in Healthcare During COVID-19 Pandemic: Rapid Scoping Review. Front. Glob. Womens Health.

[36] World Health Organization (WHO) (2019). Delivered by Women, Led by Men: A Gender and Equity Analysis of the Global Health and Social Workforce. Hum. Resour. Health Obs. Ser..

[37] Shinde V.V. (2019). Relationship of Body Mass Index to Job Stress and Eating Behaviour in Health Care Professionals-an Observational Study. Obes. Med..

[38] Olaleye T.T., Christianson T.M., Hoot T.J. (2022). Nurse Burnout and Resiliency in Critical Care Nurses: A Scoping Review. Int. J. Afr. Nurs. Sci..

[39] Han P., Duan X., Wang L., Zhu X., Jiang J. (2022). Stress and Coping Experience in Nurse Residency Programs for New Graduate Nurses: A Qualitative Meta-Synthesis. Front. Public Health.

[40] Babkair L.A., Alaamri M., Tunsi A., Alhofaian A., Alsulami N.M., Hakami M.Y., Alshehri N.A., Alsulami W.M. (2024). Stress Level and Coping Strategies among Intensive Care Nurses: A Cross-Sectional Study. J. Educ. Health Promot..

[41] Observatório de Sergipe Censo 2022: 62% Da População Sergipana Se Identifica Como Parda. https://observatorio.se.gov.br/censo-2022-62-da-populacao-sergipana-se-identifica-como-parda/.

[42] Santos M.E.R.d., Sousa A.L.N.d. (2025). Narrativas Escreviventes de Enfermeiras Negras No Enfrentamento Ao Racismo e Sexismo. Saúde E Soc..

[43] Tembo A.C., Moorley C. (2025). Reckoning With Racism in Nursing: Towards Structural Transformation and Epistemic Justice. J. Adv. Nurs..

[44] Hamed S., Bradby H., Ahlberg B.M., Thapar-Björkert S. (2022). Racism in Healthcare: A Scoping Review. BMC Public Health.

[45] Pan American Health Organization (2022). The COVID-19 HEalth CaRe WOrkErs Study (HEROES): Regional Report from the Americas.

[46] Rodrigues C.C.F.M., Santos V.E.P., Tourinho F. (2016). Estresse—Normal Ou Patológico?. Saúde Transform. Soc. Health Soc. Change.

[47] Nascimento J.C.P., Costa T.M.d.S., Sarmento S.D.G., Santos K.V.G.d., Dantas J.K.d.S., Queiroz C.G., Dantas D.V., Dantas R.A.N. (2022). Análise Do Transtorno Do Estresse Pós-Traumático Em Profissionais Emergencistas. Acta Paul. Enferm..

[48] Zhou H., Liao H., Huang Y., Lin Q., Wang X., Li H., Wu F., Yang S. (2024). Moral Distress in Pediatric Nurses: A Scoping Review Protocol. PLoS ONE.

[49] Bruyneel A., Dello S., Dauvergne J.E., Kohnen D., Sermeus W. (2025). Prevalence and Risk Factors for Burnout, Missed Nursing Care, and Intention-to-Leave the Job among Intensive Care Unit and General Ward Nurses: A Cross-Sectional Study across Six European Countries in the COVID-19 Era. Intensive Crit. Care Nurs..

[50] Silva A.K.L.d., Bendassolli P.F. (2019). Coletivos de Trabalho e a Produção de Saúde Dos Ofícios. Psicol. Estud..

[51] Yaribeygi H., Panahi Y., Sahraei H., Johnston T.P., Sahebkar A. (2017). The Impact of Stress on Body Function: A Review. EXCLI J..

